# Spatial Orientation in Japanese Quails (*Coturnix coturnix japonica*)

**DOI:** 10.1371/journal.pone.0028202

**Published:** 2011-12-07

**Authors:** Tim Ruploh, Agnieszka Kazek, Hans-Joachim Bischof

**Affiliations:** 1 Lehrstuhl Verhaltensforschung, Universität Bielefeld, Bielefeld, Nordrhein-Westfalen, Germany; 2 Institut für Biologie und Umweltwissenschaften, AG Neurosensorik, Universität Oldenburg, Oldenburg, Niedersachsen, Germany; University of Lethbridge, Canada

## Abstract

Finding a given location can be based on a variety of strategies, for example on the estimation of spatial relations between landmarks, called *spatial orientation*. In galliform birds, spatial orientation has been demonstrated convincingly in very young domestic chicks. We wanted to know whether adult Japanese quails (*Coturnix coturnix japonica*) without food deprivation are also able to use spatial orientation. The quails had to learn the relation of a food location with four conspicuous landmarks which were placed in the corners of a square shaped arena. They were trained to find mealworms in three adjacent food cups in a circle of 20 such cups. The rewarded feeders were located during training between the same two landmarks each of which showed a distinct pattern. When the birds had learned the task, all landmarks were displaced clockwise by 90 degrees. When tested in the new situation, all birds redirected their choices with respect to the landmark shift. In subsequent tests, however, the previously correct position was also chosen. According to our results, quails are using conspicuous landmarks as a first choice for orientation. The orientation towards the previously rewarded location, however, indicates that the neuronal representation of space which is used by the birds also includes more fine grain, less conspicuous cues, which are probably also taken into account in uncertain situations. We also presume that the rare orientation towards never rewarded feeders may be due to a foraging strategy instead of being mistakes.

## Introduction

Orientation in space is an indispensable skill for every mobile animal. It can be based on a variety of different cues. Among others, the sun compass, the earth magnetic field, the stellar constellations, or the polarisation pattern of the sky can be used for finding the correct course [1]. Such cues provide a reference frame particularly useful for navigation over long distances. However, because only a direction but not a distance is given, it is difficult to identify a particular location. Animals therefore often rely on landmarks, that is on conspicuous objects scattered around the landscape, probably in addition to the previously mentioned orientation mechanisms. The ability to reach a desired goal by using the spatial arrangement of landmarks including distance, geometric information, individual characteristics of landmarks, and the goal location is called spatial orientation. By learning relations between the goal and one or several landmarks, an animal acquires a “cognitive map” or “neuronal representation” of the more or less complex spatial structure of the environment. The cognitive map is based on previously acquired knowledge about the environment and is updated by each travel to another destination [2], [3]. By such neuronal representation, the animal is able to locate the desired goal independent of its own position. This has been shown in numerous studies on spatial orientation in a large number of animals [4]–[8].

An ongoing debate concerns the use of so called local and distant/global cues. Although there is not a commonly accepted definition of the two terms, most authors are using “local” for cues which are within the experimental space of the experiment [5]. Some authors include traits of the goal location itself into local cues [9], [10]; others don't [11], [12]. The term “distant” cues is used for those that are outside of the experimental space [5], [13]. This is operationally acceptable but not applicable for natural settings. To our knowledge, there is at present no solution of this problem. It might be feasible to attribute cues as local if they are so near to the goal that the animal has the chance to estimate distances between the landmarks and between landmarks and goal, while landmarks may be defined as distant ones which are so far away that distance estimation is difficult. This definition is still not really exact, but it could be applied to experiments in natural settings with no distinct borders of the animal's environment.

In birds, many studies have dealt with orientation of two passerine families, the *Corvidae*, including nutcrackers and jays, and the *Paridae* comprising chickadees and tits. Both are famous for their ability to cache numerous food items at widely dispersed places and to recache it with great precision by using the spatial arrangement of landmarks [14]–[16].

Some experiments indicate that food storing animals are depending mostly on distant cues because local cue traits may be uncertain in a changing environment [10], [16]. Non-storing birds, in contrast, have been shown to attend to both kinds of cues to find their goal, with a preference for local ones [12], [16], [17]. As in most cases, this dichotomy turned out to be too simple. Food storing birds are also using local cues when these are conspicuous and reliable [18], [19]. Obviously, birds are using all kinds of cues dependent on the situation [9], [20]. Because local cues are in general easier to spot [21] and allow more precise orientation by distance estimation and use of geometrical calculations [22]–[24], they are preferred over distant ones if they are reliable enough over time [9].

Among galliform birds, it is only the domestic chick which is examined for its spatial orientation skills. Vallortigara and colleagues developed an experimental design where the birds had to find hidden food in the center of a square shaped arena. They showed that the birds were able to locate the hidden location by a single beacon [25], [26] as well as by arrays of local landmarks [21]. However, in most cases, the birds were using just the distance from a single landmark and/or from the area walls as well as other geometrical information instead of the relation of several landmarks [22]. In addition, there was evidence that the left brain hemisphere was encoding predominantly local cues and absolute metric information, while the right one encoded relations between landmarks and the geometry of the enclosure [25]–[27].

The experimental design of our study differed from that used by Vallortigara and colleagues in that we did not train our animals to a central location. Although this training to the center has gained excellent results, it is not fully comparable to most of the other avian studies which used peripheral goal locations. In contrast to most other studies, we did not deprive the birds from food but used mealworms as tidbits for the training to test whether such experiments are also possible without hunger stress for the animals. One of the reasons for using Japanese quails was our belief that comparative experiments strongly enhance the universal validity of scientific findings. More important, however, was the idea that there might be differences between very young animals as used in the domestic chick studies and adult birds [28]. Because of the size difference between adult domestic chicks and quails, we preferred the latter because small birds are much easier to keep and to handle in experiments. Our experiments may therefore lay the grounds for a more detailed analysis of the orientation skills of adult galliform birds. They were initially not intended to extend our knowledge about subtle details of the already known facts. It turned out, however, that some of our findings were supportive of previous findings; others did not fit into the existing framework.

## Materials and Methods

### Ethics statement

Behavioral studies without food or social restrictions are not regulated by the German animal protection law and do not require special approval.

Ten adult Japanese quails (*Coturnix coturnix japonica*, 3 to 5 month old, 5m/5f) were used for this study. The birds were kept either individually or in groups of up to 4 animals, depending on the level of aggression against conspecifics, at a light cycle of 14 L ∶ 10 D. Independent of the number of birds, rectangular arenas (200×85×50 cm) were used for housing. Water and food were provided *ad libitum*. Light was on at 7:30 a.m., daily experiments started at 9:00 a.m.

**Figure 1:**
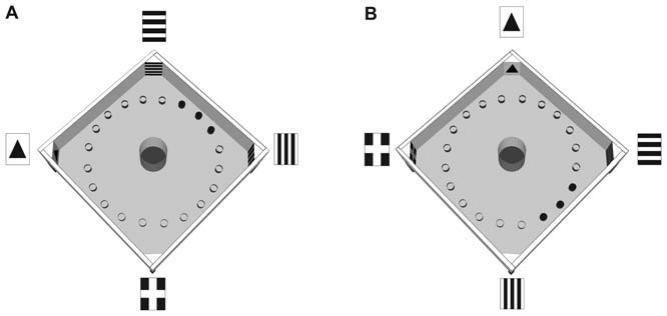
Experimental arena. In the arena (150×150×50 cm) 20 identical food cups were arranged in a circle and four distinct visual landmarks placed in the corners. The central, transparent starting cylinder could be retracted into the floor. Filled circles represent baited food cups. Quails were trained to find food between two specific landmarks (A). In the test, landmarks and positions of baited food cups were shifted 90° clockwise (B).

A squared arena (1.50 m×1.50 m) with 50 cm high walls was constructed from white laminated chipboard (fig. 1) and placed on a table. In the center, a cylindric start box made from plexiglass tube (diameter: 20 cm, height: 50 cm) was installed. The wall of the start box was constructed to be sunk into the arena's floor by an elevator mechanism controlled from the adjacent room. Twenty identical grey food cups (diameter: 4 cm, height: 4 cm) were arranged in a circle (diameter: 120 cm) with a distance of 8.5 cm/18° to each other. A two-dimensional landmark with a distinct pattern (vertical stripes, horizontal stripes, cross or triangle) was placed in each corner (fig. 1). The spatial relation of the four landmark patterns was kept constant throughout the experiment.

The cups were filled with freshly frozen and defrosted mealworms. A grid cover hindered the birds to access the food in all except three baited cups. Fine wooden shavings covered the floor and were also put inside the cups to hide the mealworms. These wooden shavings were rearranged after each trial.

The entire setup was placed in the center of a homogeneously illuminated white plastic pavilion (3 m×3 m×3 m) to eliminate uncontrolled external visual cues. A video camera chip was mounted at the ceiling of the pavilion by which all experiments were recorded.

At the beginning of each trial, the experimental animal was brought into the pavilion using randomly one of the four entrances at each side of the pavilion and was placed into the start box. The experimenter left the pavilion and, one minute later, removed the start box wall by the remote system from the adjacent room.

Training comprised several steps. In the pre-training phase, only a single accessible food cup with mealworms not covered by wooden shavings was placed at some random position within the arena to familiarize the birds with the setup and the food cups. Pre-training was continued until the birds did not any longer show any signs of stress when they were placed in the start box. The next step was to cover the mealworms with wooden shavings.

When the bird had learned to find the covered mealworms, training for the spatial memory test began. This was again performed in two steps because pilot experiments showed that the birds were not able to learn the task in only one step as it is used in most other training experiments. Instead of the one randomly placed food cup, the birds were now exposed to the circle of 20 food cups. Each of the cups contained food covered by a grid and wooden shaving except three adjacent ones where the grid (but not the wooden shaving) was replaced so that the birds had access to the food. The three adjacent food cups were placed at one of two positions depending on the group to which the subjects belonged. Animals were allowed to probe any cup until they finally found a cup where food was accessible. For half of the birds, the cup at zero degrees and the two adjacent cups between the vertically and the horizontally striped landmarks (fig. 1) were accessible. For the other birds, the food at the cup at 180 degrees and the two adjacent cups (flanked by the “cross” and the “triangle” landmark) was accessible.

When the birds appeared to perform above chance level, the final training was started. From that time on, animals were only permitted to choose once. If this first choice was correct, the quail was allowed to feed before it was removed from the test arena. If it was incorrect, a period of 20 s darkness followed before the quail was removed without access to the mealworms. Each subject was trained daily until it succeeded to make three correct choices. No more than 12 trials were made every day. The learning criterion was reached when the quails made 6 correct choices in a sequence of 10 (binomial test: test value = 0.15, p<0.001).

To test whether the quails located the accessible food using the landmarks, these and also the sites with access to the food were shifted by 90° clockwise. The trained quails were then tested ten times under this shifted condition. Five of the test trials were performed immediately after the learning criterion was reached, and five at the following day.

Because there was no significant difference between the “zero” and the “180 degree” group, both were lumped together and normalized.

The circularly distributed data were processed and tested using Oriana (Kovach Computing Services, Pentraeth, Wales, U.K.), a program designed for circular statistics which adapts the calculations according to the use of discrete or continuous data. The mean of the choice directions of each individual quail is described by a vector; its length r depends on the variance of the data, being large when the variance is low and small if it is high. Rayleigh's Uniformity Test was used to test the normal distribution of the data. A V-Test was then applied to examine whether the measured mean coincided with an expected one. The theoretically expected means were 0 degrees in the training and 90 degrees in the test trials.

Other statistical analyses were made using SPSS 19.0. Choice scores (s. Results) were compared using the Friedman Test. For pair-wise posthoc comparisons, a one-sided Wilcoxon Test with Bonferroni correction was used.

## Results

**Figure 2:**
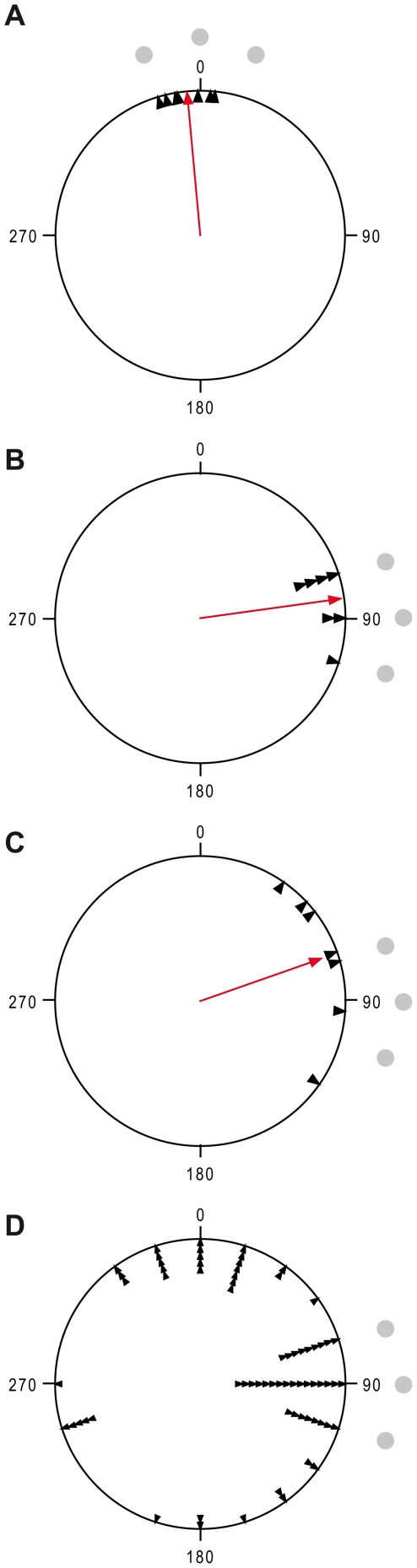
Results. Black dots represent baited positions. A) Small arrows represent individual means calculated from the last ten training trials B) Small arrows depict individual choice directions in the first test trial. C) Small arrows show individual means of choice directions calculated from the ten test trials. D) Small arrows point to individual single choices in all ten test trials. A–C) The centrally based arrow depicts the mean vector calculated from the individual values.

Seven quails reached the defined learning criterion (6 correct choices within 10 consecutive trials). The number of training trials to reach the criterion was very different between animals and ranged from 15 to 46. No relation of the number of training trials with the test results could be observed. Individual direction means (computed from the last ten training trials) ranged from 344° to 16° (Figure 2a, N = 7, overall mean vector = 355°, r = 0.99, Rayleigh's Uniformity Test: Z = 6.861, p<0.0001. V-Test with expected mean of 0°: V = 0.986, u = 3.689, p<0.0001). The remaining three quails were excluded from the experiment because the learning criterion was not reached.

In the first test trial after the 90° landmark shift, all quails followed the landmark rotation and chose one of the three new correct positions around 90 degrees (Figure 2b, N = 7, overall mean vector = 82°, r = 0.97, Rayleigh's Uniformity Test, Z = 6.642, p<0.0001. V-Test with expected mean of 90°: V = 0.965, u = 3.611, p<0.0001).

The next nine test trials gained less consistent results. In some cases, other than the correct positions were chosen, resulting in individual means ranging from 36° to 125° (Figure 2c). The mean vector had shifted by 11 degrees compared with that of the first trial, and the vector length was also slightly reduced (N = 7, mean vector = 71°, r = 0.88, Rayleigh's Uniformity Test: Z = 5.437, p = 0.001, V-Test with expected mean of 90°: V = 0.832, u = 3.113, p<0.001).

The reason for this difference is depicted in figure 2d. While the birds in the first test trial exclusively chose the new position as defined by the rotated landmarks, each bird made a number of choices to the old correct position. Oriana treated this bimodal distribution as a unimodal one. The shift away from the new theoretical correct position was thus an artifact caused by the bimodality of the data.

**Figure 3:**
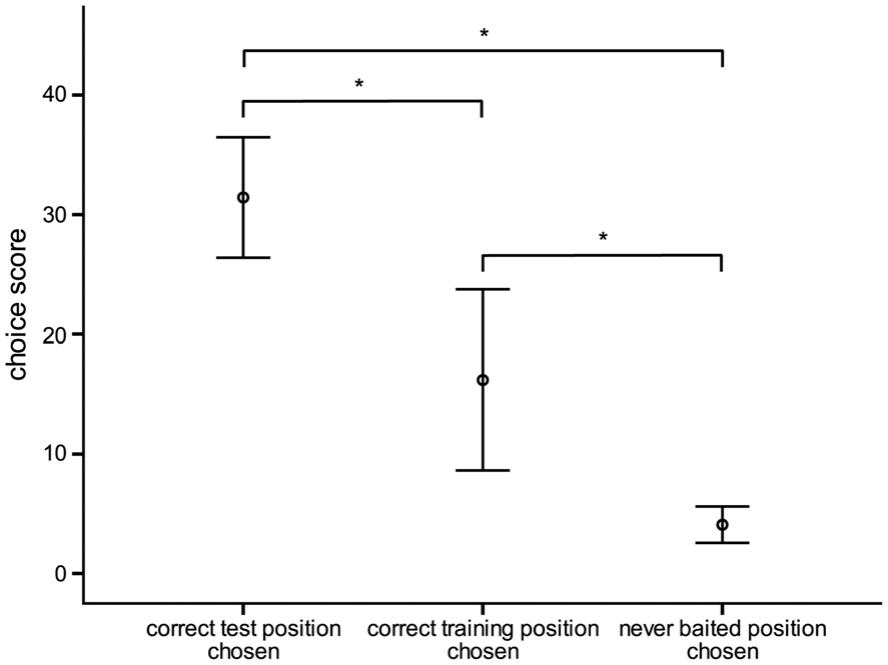
Choice scores. Choice scores calculated from all ten test trials (N = 7, means ± SD). In the test phase, quails could choose between three alternatives. First, one of the actually accessible cups, second, a cup that was baited in the training sessions, third, a cup that was never baited before. For calculation of choice scores and statistics see text.

In the test phase, the birds had three alternatives, the first being a choice correct with respect to the landmarks, the second to choose the cups which were correct in the training condition and third, the chosen cup was not correct either during training or during test trials. To determine which of the three alternatives was preferred, we corrected the measurements according to the probability to choose any of these alternatives. These probabilities were 0.7 (14/20) for a never correct cup, 0.15 (3/20) for the choice of one of the cups correct at the training trials and also 0.15 for the choice of one of the correct cups at the test trials. The calculated score was highest for choosing one of the positions which were indeed the correct ones at the test (30.43±5.04; mean ± SD), followed by the score for choosing a previously correct position (16.19±7.56; mean ± SD). As depicted in figure 3, the score for choosing a cup which was never correct was (4.08±1.53; mean ± SD). The differences were significant (Friedmann test, n = 7, Chi^2^ = 11.630, p = 0.003.), and this was true for all pairwise comparisons as revealed by a posthoc Wilcoxon test (N = 7, correct test vs. correct training: Z = −2.207, p = 0.04; correct test vs. never baited: Z = −2.371, p = 0.03; correct training vs. never baited: Z = −2.201, p = 0.04).

## Discussion

The present study clearly demonstrates that Japanese quails are able to use landmarks to locate the position of a food source. The birds oriented reliably towards food cups that had a constant spatial relation to four distinct landmarks. When the four landmarks were simultaneously displaced clockwise by 90°, all subjects changed their orientation accordingly in the first test trial. Continuing the tests until each bird had performed 10 trials showed that after an initially uniform response, the birds also chose the orientation which had been learned in the training phase. Obviously, the first choice after the landmark displacement was guided by these conspicuous cues. In subsequent trials, however, additional cues which had not been displaced were also used for orientation. Whether these were tiny markings like scratches on the walls which were not identified when designing the experiment or more distant landmarks outside the arena cannot be decided. At least theoretically, it can also not be excluded that some acoustic cues from external sources or even the earth magnetic field caused the birds to split their choices.

Interestingly, the birds did not choose an intermediate orientation as it has been shown in the pigeon [29]. The old and the new goal directions have thus been kept separately. This is obviously the more appropriate solution because a compromise direction under such experimental conditions does not make sense. Pigeons may use this compromise because it makes sense if there is, for example, a mismatch between the sun compass and the magnetic compass [30]. Alternatively, the quails may have learned in our experiments that there is never food directly at the landmarks, thus there may be no real difference between the pigeon and the quail strategies.

In any case, our results indicate that the spatial map of the quails is not only based on a few conspicuous landmarks, but may contain a quite fine grain description of the environment including also a variety of low-key cues. As already mentioned above, the quails may have used the features of the map depending on the situation, trying the most conspicuous landmarks first and then going on to others if there were indications of inconsistency between the highly and less conspicuous cues.

This arrangement, however, could be used to examine whether quails, as it has been shown in the chicken, may use distances to landmarks for the estimation of the correct feeder. If so, each individual chick should have shown a preference for one of the three feeders in the course of the test trials. This was not the case. Because, however, the birds in the pilot studies did not perform well with only one feeder, one cannot decide whether the lack of a preference for one feeder indicates that the birds did not use distances, or whether this was just due to impreciseness. Because the birds did not make errors by accessing the trays directly flanking the three accessible ones, the most plausible explanation might be an acquired rule like “food is between the two conspicuous landmarks” or “food is left (right) from one conspicuous landmark”. The latter explanation fits the results obtained in the domestic chicks [22] which also favour the use of only one landmark.

Quails accidentally chose incorrect, strongly deviating food locations even after extensive training. These could be errors because of the difficulty of the task, because the probability to choose the correct feeders by chance was quite low (3/20). However, because all birds were correct at the first test trial, and the predominant “mistakes” in the subsequent trials were also “correct” because it was the original training direction, another explanation may be more plausible. According to optimal foraging theory [31], [32], animals instead of emptying one food source completely and then turning to another, should keep track of the content of other possible food sources by incidental visits. The frequency of these incidental visits, which are counted in a learning design like this as errors, depends on the hunger level of the animal. The animals in our experiment were not food deprived, and thus the motivation to sample other possible food sources might have been higher than in experiments with food deprived animals. Whether our use of mealworms as tidbits for training also enhanced the tendency to inspect other than the rewarded trays cannot be decided. We used this food because mealworms induced even in fully fed quails a run towards the caretaker when he presented the worms. Obviously, this is a very much preferred food for the birds.

Concerning age effects, we had the impression that the readiness of the birds to learn the task decreased with age in contrast to the notion of Meinecke [28] who claimed that very young quail chicks were inferior concerning aspects of one trial avoidance tasks. Such decrease is plausible because quails are known for very fast aging (life span is between 2 and 3 years, adulthood reached with 64 days) and clear aging effects [33]. Because we also did not use food deprivation, it is not clear which of these two factors was responsible for the slowness of learning.

Taken together, our results indicate that adult quails are still able to learn spatial orientation tasks. They are preferably using conspicuous landmarks, but are able to include less obvious and probably more distant cues in case the situation becomes unclear. This can be taken as a hint for the use of an internal representation of the spatial environment in addition to geometrical parameters from single nearby landmarks.

## References

[1] Schöne H (1980). Orientierung im Raum: Formen und Mechanismen der Lenkung des Verhaltens im Raum bei Tier und Mensch.

[2] Bennett ATD (1996). Do animals have cognitive maps?. J Exp Biol.

[3] Gallistel CR (1989). Animal cognition, the representation of space, time and number.. Ann Review Psychol.

[4] O'Keefe J, Nadel L (1978). The Hippocampus as a Cognitive Map.

[5] Morris RGM (1981). Spatial localization does not require the presence of local cues.. Learn Motiv.

[6] Gagliardo A, Ioale P, Bingman VP (1999). Homing in pigeons. The role of the hippocampal formation in the representation of landmarks used for navigation.. J Neurosci.

[7] Rodriguez F, Lopez JC, Vargas JP, Gomez Y, Broglio C (2002). Conservation of spatial memory function in the pallial forebrain of reptiles and ray-finned fishes.. J Neurosci.

[8] Dyer FC, Gould JL (1981). Honey bee orientation: a backup system for cloudy days.. Science.

[9] LaDage LD, Roth TC, Fox RA, Pravosudov VV (2009). Flexible cue use in food-storing birds.. Anim Cogn.

[10] Brodbeck DR (1994). Memory for spatial and local Cues - A comparison of a storing and a nonstoring species.. Animal Learning & Behavior.

[11] Watanabe S, Mayer U, Bischof HJ (2011). Visual Wulst analyses “where” and entopallium analyses “what” in the zebra finch visual system.. Behav Brain Res.

[12] Mayer U, Bischof HJ (2011). Brain activation pattern depending on the strategy chosen by zebra finches to solve an orientation task.. Exp Biol.

[13] Spetch ML, Edwards CA (1988). Pigeons', *Columbia livia*, use of global and local cues for spatial memory.. Anim Behav.

[14] Vander Wall SB, Balda RP (1977). Coadaptations of the Clark's Nutcracker and the pinon pine for efficient seed harvest and dispersal.. Ecol Monogr.

[15] Pravosudov VV (1985). Search for and storage of food by *P. cinctus lapponicus* and *P. montanus borealis* (Paridae).. Zool Zh.

[16] Clayton NS, Krebs JR (1994). Memory for spatial and object-specific cues in food-storing and non-storing birds.. J Comp Physiol A.

[17] Brodbeck DR, Shettleworth SJ (1995). Matching location and color of a compound stimulus: comparison of a food-storing and a nonstoring bird species.. J Exp Psychol Anim B.

[18] Hodgson ZG, Healy SD (2005). Preference for spatial cues in a non-storing songbird species.. Anim Cogn.

[19] Feenders G, Smulders TV (2011). Magpies can use local cues to retrieve their food caches.. Anim Cogn.

[20] Herborn K, Alexander L, Arnold KE (2011). Colour cues or spatial cues? Context-dependent preferences in the European greenfinch (*Carduelis chloris*).. Anim Cogn.

[21] Della Chiesa A, Pecchia T, Tommasi L, Vallortigara G (2006). Multiple landmarks, the encoding of environmental geometry and the spatial logics of a dual brain.. Anim Cogn.

[22] Della Chiesa A, Speranza M, Tommasi L, Vallortigara G (2006). Spatial cognition based in geometry and landmarks in the domestic chick (*Gallus gallus*).. Behav Brain Res.

[23] Spetch ML, Cheng K, MacDonald SE (1996). Learning the configuration of a landmark array: I. touch-screen study with pigeons and humans.. J Comp Psych.

[24] Pecchia T, Vallortigara G (2010). View-based strategy for orientation by geometry.. J Exp Biol.

[25] Tommasi L, Vallortigara G (2001). Encoding of geometric and landmark information in the left and right hemispheres of the avian brain.. Behav Neurosci.

[26] Tommasi L, Vallortigara G (2004). Hemispheric processing of landmark and geometric information in male and female domestic chicks (*Gallus gallus*).. Behav Brain Res.

[27] Tommasi L, Gagliardo A, Andrew RJ, Vallortigara G (2003). Separate processing mechanisms for encoding of geometric and landmark information in the avian hippocampus.. Europ J Neurosci.

[28] Meinecke RO (1974). Retention of One-Trial Learning in Neonate, Young Adult, and Aged Japanese Quail.. J Gerontol.

[29] Cheng K (1988). Some psychophysics of the pigeon's use of landmarks.. J Comp Physiol A.

[30] Wiltschko R, Kumpfmüller R, Muth R, Wiltschko W (1994). Pigeon homing: the effect of a clock-shift is often smaller than predicted.. Behav Ecol Sociobiol.

[31] MacArthur RH, Pianka ER (1966). On the optimal use of a patchy environment.. Am Nat.

[32] Inglis IR, Langton S, Forkman B, Lazarus J (2001). An information primacy model of exploratory and foraging behaviour.. Anim Behav.

[33] Ottinger MA (2001). Quail and other short-lived birds.. Exp Gerontol.

